# Determinants of knowledge of HIV status in South Africa: results from a population-based HIV survey

**DOI:** 10.1186/1471-2458-9-174

**Published:** 2009-06-05

**Authors:** Karl Peltzer, Gladys Matseke, Thembile Mzolo, Mmapaseka Majaja

**Affiliations:** 1 Health Promotion Research Unit, Social Aspect of HIV/AIDS and Health, Human Sciences Research Council, Pretoria, South Africa; 2 Department of Psychology, University of the Free State, Bloemfontein, South Africa

## Abstract

**Background:**

Over 30% of women and men in the South African national HIV household of 2005 indicated that they had previously been tested for HIV (of which 91% were aware of their test results). This paper seeks to describe the associations between socio-demographic, behavioural and social characteristics and knowledge of HIV status among a nationally representative population in South Africa.

**Methods:**

A multistage probability sample involving 16395 male and female respondents, aged 15 years or older was selected. The sample was representative of the South African population by age, race, province and type of living area, e.g. urban formal, urban informal, etc. Respondents were interviewed on HIV knowledge, perceptions and behaviour and provided blood for research HIV testing. Bivariate and multivariate logistic regression was used to identify socio-demographic, social and behavioural factors associated with knowledge of HIV status.

**Results:**

From the total sample 27.6% ever and 7.8% knew their HIV status in the past 12 months. In multivariate analyses being female, the age group 25 to 34 years old, other than African Black population group (White, Coloured, Asian), higher educational level, being employed, urban residence, awareness of a place nearby where one could be tested for HIV, impact of HIV on the household and having had two of more sexual partners in the past year were associated with knowledge of HIV status. Among HIV positive persons awareness of a place nearby where one could be tested for HIV and impact of HIV on the household were associated knowledge of HIV status, and among HIV negative persons HIV risk behaviour (multiple partners, no condom use), awareness of a place nearby where one could be tested for HIV, higher knowledge score on HIV and knowledge of serodiscordance were associated knowledge of HIV status.

**Conclusion:**

Education about HIV/AIDS and access to HIV counselling and testing (HCT) in rural areas, in particular among the Black African population group needs to be improved, in order to enhance the uptake of HIV counselling and testing services, an essential step for the initiation of treatment.

## Background

HIV Counselling and Testing (HCT) is an entry point to both prevention and treatment. People have to know their HIV sero-status to enable them to seek appropriate treatment as well as enable them to choose prevention strategies. It has become increasingly important in the global response to HIV/AIDS. HCT services need to be available and accessible to the entire public to enable their easy utilization. Although most South Africans are aware that HCT services are available, only one in five people in South Africa who know about HCT have been tested for HIV [1]. Bunnell [2] found in a cross-sectional and nationally representative study (2004–2005 Uganda HIV/AIDS Sero-Behavioral Survey) that 21% of adults knew their HIV status. Among countries in Southern Africa the percentage who took an HIV test in the last 12 months and who know their results ranges between 2% in Mozambique to 20% in Botswana [3], and in the US 10.4% [4].

Various factors have been identified for the low uptake of HCT: (1) socio-economic factors such as age [5-9], marital status [8], educational level [6,10,11], occupation [7], household wealth [10], and area of residence [8]; (2) social factors such as fear of unsolicited disclosure, fear of stigma and discrimination [1,6,7,9,12,13], client-counsellor dynamics including lack of confidentiality [11,14,15]; (3) proximity and access to VCT site [6,9,12,16,17]; (4) HIV knowledge including prior knowledge of VCT sites and HIV risk perception and HIV risk behaviour [7,8,10,11,18,19]; and (5) health status [5,7,8].

Various studies in South Africa have also shown that the uptake of HCT also differs depending on the counselling and testing model used, the use of HCT testing kits, other than rapid testing kits across different populations of high HIV prevalence [20-23]. There is lack of information on determinants of knowledge of HIV status in South Africa. Therefore, this study investigates the determinants of knowledge of HIV status in South Africa using secondary data analysis from a national population-based HIV survey.

## Methods

### Sample and procedure

The survey targeted all persons over 2 years of age living in South Africa and residing in homes, i.e. excluding individuals living in educational institutions, old-age homes, hospitals and uniformed service barracks but including those living in hostels. The survey applied a multi-stage stratified sampling approach based on a master sample consisting of 1 000 enumerator areas (EAs) used by Statistics South Africa for the national census in 2001. Three persons in each household were potentially eligible to be selected for the survey; however only one was selected from each of the age groups 2–14 years, 15–24 years, and 25 years and older. The sample included in this analysis includes the age group 15 years and above (range 15 to 96) 16395 (59.1% Black Africans, 18.4% Coloureds, 11.7% Whites and 10.8% Indian or Asians) of which 12 032 were interviewed and tested for HIV and 4363 who were interviewed but not tested for HIV. Linked anonymous HIV testing was performed using dried blood spot (DBS) specimens. Socio-demographic and behavioural information was collected with questionnaires administered by trained fieldworkers [5].

Ethical approval for conducting the study was obtained from the Human Sciences Research Council's Ethics Committee (Application Number REC5/24/04). Informed consent was obtained separately for agreeing to participate in the interview and for providing a specimen for HIV testing.

### Measures

HIV antibody testing: Using DBS spots all samples were first tested with Vironostika HIV-1 Uniform II Plus O assay (bioMerieux); all HIV positive samples were retested with a second ELISA test (Vitros ECI, Ortho Clinical Diagnostics) (Shisana et al., 2005).

The questionnaire included demographic variables such as age, sex, formal education completed, marital status and socioeconomic status.

HIV testing history: The survey included questions concerning history of HIV antibody testing. These measures were used to classify participants into groups based on whether they had been tested for HIV and knew their results. Participants who reported having been tested for HIV indicated their HIV awareness status of their most recent test, or that they did not know the results.

HIV risk behaviour history: To assess HIV risk history, participants indicated the number of sex partners they had in the previous 12 months, had symptoms of a sexually transmitted infection (STI), and whether they had ever used a condom, a condom with their last sexual partner and their last sexual non-regular partner. All responses were dichotomous indicating the occurrence or non-occurrence of each risk factor.

HIV knowledge: A 7-item HIV knowledge test was used, e.g. Is it possible to transmit HIV through unprotected sex? Response options were yes, no, does not know. Responses were scored for the number of correct responses; with don't know responses scored incorrect, range 0–6. Scores were coded into three levels low = 5 correct responses; medium = 6 correct responses, and high = 7 correct responses. Cronbach's alpha for the HIV knowledge index was .70 for this sample.

ARV knowledge was assessed with one item: "Have you ever heard about new drug treatments for people with AIDS called antiretrovirals or ARVs?" Response option was "Yes" or "No".

HIV impact: Participants responded to three HIV impact items, ever anyone in the household ever been diagnosed with HIV-AIDS, is there a person in the household who is bed-ridden with an AIDS related illness and past year occurrence of AIDS-related death of household member. Response options were "yes or no"; any yes was scored with "1".

AIDS stigma attitudes: Five AIDS stigma items were adapted from previous research and developed for use in South Africa, e.g. "I would be willing to care for a family member with AIDS". Response options were, yes, no, do not know; "no and Do not know were coded one and yes coded "0". A total score was calculated, (range 0–5), and coded into three levels 0= scores low AIDS stigma, medium 1–2 and 3–5 scores high AIDS stigma. Cronbach's alpha for the AIDS stigma index was .62 for this sample.

Demographic variables included sex, age, marital status, population group (Black African, White, Coloured, Indian or Asian), formal education, employment status and place of residence.

### Data analysis

Data analysis was performed using STATA software version 10.0 (Stata Corporation, College Station, Texas, USA). The analysis in STATA took into account the multilevel stratified cluster sample design of the study. We obtained frequencies as estimation of prevalence of knowledge of HIV status. We also conducted logistic regression analysis to estimate the association between relevant predictor variables and knowledge of HIV status. The predictor variables were identified from the literature as possible factors that may be associated with knowledge of HIV status [5-19]. We report unadjusted odds ratios for selected predictor variables (sex, age, marital status, formal education, population group, employment status, geolocality, awareness of HIV test site, HIV knowledge, knowledge of ARVs, knowledge of HIV serodiscordance, impact of HIV, HIV risk perception, HIV stigma attitudes, HIV status, history of STI symptoms, number of sexual partners in the past year, no condom use at last sex, no condom use with last non-regular partner) while considering knowledge of HIV status as a dependent variable, and knowledge of HIV status separately for HIV positive and HIV negative as dependent variables. We therefore report results of adjusted odds ratios for the factors, having controlled for factors as significant in the bivariate analysis. The dependent variable was knowledge of HIV status, and the independent variables were factors which significantly increased knowledge of HIV status in the bivariate analysis. In the analysis, weighted percentages are reported. The reported sample size refers to the sample that was asked the target question. The two-sided 95% confidence intervals are reported. The p-value less or equal to 5% is used to indicate statistical significance. Both the reported 95% confidence intervals and the p-value are adjusted for the multi-stage stratified cluster sample design of the study.

## Results

From the total sample of 16395 15 years and above, 27.6% (CI = 26.5–28.7) reported to have ever had an HIV test and had received their HIV test results (knowledge of HIV status). Of those who had been tested for HIV, 38.8% had been tested within the year preceding the survey, 33.1% 1 to 2 years previously, and 28.2% more than 2 years previously; 7.8% of the total sample had taken an HIV test in the past 12 months and knew their test result.

In bivariate analyses being female, the age group 25 to 34 years old, being married or cohabitating, Grade 12 and more formal education, other than African Black population group (White, Coloured, Asian), being employed, urban residence, awareness of a place nearby where one could be tested for HIV, higher knowledge score on HIV, knowledge of serodiscordance, impact of HIV on the household, high HIV risk perception, being HIV positive (from the survey), one, two or more sexual partners in the past year and non-condom use at last sex were associated with knowledge of HIV status. In multivariate analyses being female, the age group 25 to 34 years old, other than African Black population group (White, Coloured, Asian), higher educational level, being employed, urban residence, awareness of a place nearby where one could be tested for HIV, impact of HIV on the household and two or more sexual partners in the past year were associated with knowledge of HIV status, and marital status, HIV knowledge, knowledge of serodiscordance, HIV risk perception, HIV status and no condom at last sex were no longer associated with knowledge of HIV status. A large proportion (39.5%, 38.2, 40.7) indicated that they had not heard about antiretroviral treatment (see Table 1).

**Table 1:** Bivariate and multivarivate analyses of factors associated with knowledge of HIV status

	N	Know HIV test result (%)	Crude OR (95% CI)	P-value	Adjusted OR (95% CI) (Pseudo R square = .15)	P-value
**Demographics**						

*Sex*				0.000		
Men	1588	24.6	1.00		1.00	
Women	2851	30.1	1.32 (1.18–1.48)	0.000	1.93 (1.52–2.43)	0.000

*Age*				0.000		
15–24	1065	17.7	1.00		1.00	
25–34	1164	40.8	3.20 (2.71–3.78)	0.000	1.83 (1.38–2.42)	0.000
35 and more	2210	27.6	1.77 (1.55–2.02)	0.000	1.22 (0.87–1.71)	0.259

*Marital status*				0.000		
Single	1662	22.9	1.00		1.00	
Married/cobabitating	2318	34.6	1.78 (1.58–2.01)	0.000	1.35 (1.00–1.83)	0.051
Divorced/separated/widowed	436	21.7	0.93 (0.78–1.11)	0.452	1.41 (0.95–2.10)	0.087

*Education*				0.000		
Grade 7 or less	688	16.0	1.00		1.00	
Grade 8 to 11	1509	22.6	1.53 (1.31–1.79)	0.000	0.96 (0.67–1.36)	0.800
Grade 12 and more	2231	42.3	4.34 (3.72–5.07)	0.000	1.50 (1.08–2.10)	0.016

*Population group*				0.000		
Black African	2182	23.8	1.00		1.00	
Other	2247	40.5	2.17 (1.92–2.45)	0.000	1.42 (1.10–1.84)	0.007

*Employment status*				0.000		
Not employed	2191	21.1	1.00		1.00	
Employed	2158	41.0	2.60 (2.30–2.94)	0.000	1.50 (1.18–1.91)	0.001

*Geolocality*				0.000		
Rural	872	17.8	1.00		1.00	
Urban	3567	35.0	2.49 (2.20–2.81)	0.000	1.96 (1.51–2.55)	0.000

**HIV knowledge and attitudes**						

*Aware of place nearby where one could be tested for HIV*				0.000		
No	142	4.4	1.00		1.00	
Yes	4287	33.9	11.05 (8.30–14.71)	0.000	7.86 (4.92–12.55)	0.000

*HIV knowledge*				0.000		
Low	981	20.3	1.00		1.00	
Medium	1492	29.0	1.60 (1.38–1.85)	0.000	0.91 (0.68–1.22)	0.535
High	1898	32.6	1.90 (1.65–2.17)	0.000	1.19 (0.90–1.56)	0.218

*Knowledge of ARVs*				0.897		
No	1715	27.9	1.00			
Yes	2695	27.7	0.99 (0.89–1.11)	0.897	---	

*Know of serodiscordance*				0.000		
No	1529	21.0	1.00		1.00	
Yes	2892	33.7	1.92 (1.71–2.15)	0.000	1.04 (0.83–1.31)	0.715

*Impact of HIV (household: HIV, care, death)*				0.000		
No	4180	26.7	1.00		1.00	
Yes	259	43.0	2.08 (1.66–2.60)	0.000	2.82 (1.81–4.41)	0.000

*HIV risk perception*				0.000		
Low	1476	24.3	1.00		1.00	
Medium	1451	27.3	1.00 (0.88–1.12)	0.961	0.94 (0.72–1.22)	0.635
High	1492	30.8	1.18 (1.10–1.26)	0.000	0.90 (0.67–1.20)	0.469

*AIDS stigma attitudes*				0.551		
Low	2023	32.0	1.00			
Medium	1995	25.4	1.02 (0.90–1.15)	0.735	---	
High	378	21.8	1.06 (0.88–1.28)	0.549		

**HIV status and risk behaviour**						

*HIV status*				0.000		
Negative	2782	26.4	1.00		1.00	
Postive	436	32.7	1.35 (1.13–1.62)	0.000	0.93 (0.69–1.24)	0.618

*Ever STI symptoms*				0.682		
No	3708	27.5	1.00			
Yes	173	28.7	1.06 (0.80–1.40)	0.682	---	

*Number of sexual partners in past year*				0.000		
None	1064	15.5	1.00		1.00	
One	3149	35.7	3.03 (2.67–3.44)	0.000	1.22 (0.92–1.61)	0.176
Two or more	226	32.4	2.62 (2.01–3.41)	0.000	1.65 (1.07–2.53)	0.022

*No condom use at last sex*				0.000		
No	1308	35.9	1.00		1.00	
Yes	1295	45.4	1.49 (1.24–1.78)	0.000	1.12 (0.87–1.43)	0.376

*No condom use with last non-regular partner*				0.677		
No	208	35.5	1.00			
Yes	309	37.2	1.07 (0.77–1.50)	0.677	---	

Bivariate analyses with demographic variables and knowledge of HIV status among HIV positive and negative persons separately found among HIV positive persons that higher levels of formal education, being White, Coloured or Asian, and urban residence were associated knowledge of HIV status, and among HIV negative persons being female, the age group 25 to 34 years old, being married or cohabitating, higher educational levels, being White, Coloured or Asian, urban residence and being employed were associated with knowledge of HIV status.

Multivariate analyses with demographic variables and knowledge of HIV status among HIV positive and negative persons separately found among HIV positive persons that urban residence and being White, Coloured or Asian were associated with knowledge of HIV status, and among HIV negative persons being female, the age group 25 to 34 years old, being married or cohabitating, higher educational levels, being White, Coloured or Asian, urban residence and being employed were associated with knowledge of HIV status (see Table 2).

**Table 2:** Bivariate and multivarivate analyses of demographic factors associated with knowledge of HIV status among HIV positive and negative persons

*Demographic variables*	**HIV positive**					
	N	Know HIV test result (%)	Crude OR (95% CI)	P- value	Adjusted OR (95% CI) (Pseudo R square = .04)	P-value
*Gender*				0.146		
Men	104	28.7	1.00			
Women	332	34.7	1.32 (0.91–1.91)	0.146	---	

*Age*				0.351		
15–24 yrs	109	31.3	1.00			
25–34	164	35.5	1.21 (0.79–1.85)	0.377		
35+	163	30.5	0.96 (0.63–1.47)	0.862	---	

*Marital status*				0.755		
Single	235	31.7	1.00			
Married/cohabiting	150	34.6	1.14 (0.79–1.64)	0.482		
Divorced/separated/widowed	51	31.3	0.98 (0.59–1.63)	0.932	---	

*Educational level*				0.016		
Grade 7 or less	119	26.1	1.00		1.00	
Grade 8 to 11	198	34.8	1.51 (1.01–2.24)	0.043	1.38 (0.92–2.07)	0.116
Grade 12+	118	37.8	1.72 (1.09–2.76)	0.019	1.56 (0.98–2.46)	0.059

*Population group*				0.000		
Black African	396	32.2	1.00		1.00	
Other	40	54.1	2.49 (1.41–4.41)	0.000	2.04 (1.12–3.71)	0.020

*Geolocality*				0.000		
Rural	120	23.2	1.00		1.00	
Urban	316	40.5	2.25 (1.59–3.88)	0.000	2.14 (1.50–3.04)	0.000

*Employment status*				0.579		
Not employed	296	33.2	1.00			
Employed	130	31.0	0.90 (0.63–1.29)	0.579	---	

*Demographic variables*	**HIV negative**					

*Gender*				0.001		
Men	994	24.0	1.00		1.00	
Women	1708	28.5	1.41 (1.14–1.75)	0.001	1.65 (1.37–1.97)	0.000

*Age*				0.000		
15–24 yrs	681	17.0	1.00		1.00	
25–34	701	42.0	3.88 (2.88–5.22)	0.000	2.30 (1.79–2.96)	0.000
35+	1400	26.0	2.41 (1.89–3.08)	0.000	1.36 (1.04–1.79)	0.026

*Marital status*				0.002		
Single	1934	22.1	1.00		1.00	
Married/cohabiting	1468	32.8	1.72 (1.47–2.08)	0.000	1.40 (1.09–1.78)	0.007
Divorced/separated/widowed	273	20.4	0.90 (0.72–1.14)	0.390	0.95 (0.70–1.29)	0.756

*Educational level*				0.000		
Grade 7 or less	459	14.8	1.00		1.00	
Grade 8 to 11	957	21.3	1.56 (1.29–1.88)	0.000	1.62 (1.28–2.04)	0.000
Grade 12+	1362	46.6	5.04 (4.14–6.12)	0.000	3.83 (3.04–4.81)	0.000

*Population group*				0.000		
Black African	1354	22.3	1.00		1.00	
Other	1422	39.8	2.30 (1.97–2.68)	0.000	1.21 (1.02–1.44)	0.028

*Geolocality*				0.000		
Rural	560	17.3	1.00		1.00	
Urban	2222	33.5	2.41 (2.06–2.81)	0.000	1.58 (1.31–1.91)	0.000

*Employment status*				0.000		
Not employed	1393	19.9	1.00		1.00	
Employed	1330	40.7	2.76 (2.35–3.25)	0.000	1.93 (1.58–2.35)	0.000

Bivariate analyses with behavioural factors (adjusted for sex and age) and knowledge of HIV status found among HIV positive persons no associations between behavioural risk factors and knowledge of HIV status, and among HIV negative persons bivariate and multivariate analyses (adjusted for sex and age) found that one, two or more sexual partners in the past 12 month and no condom use at last sex were associated knowledge of HIV status (see Table 3).

**Table 3:** Bivariate and multivarivate analyses of behavioural factors associated with knowledge of HIV status among HIV positive and negative persons

**Behavioural factors**	**HIV positive**					
	N	Know HIV test result (%)	Crude OR (95% CI)	P-value	Adjusted OR (95% CI)^1 ^(Pseudo R square = .02)	P-value

*Number of sexual partners (past year)*				0.532		
None	109	30.5	1.00		---	
One	295	33.6	1.15 (0.78–1.70)	0.471		
Two or more	32	32.6	1.10 (0.55–2.20)	0.782		

*No condom use at last sex*				0.383		
No	176	40.8	1.00		---	
Yes	98	35.9	0.81 (0.51–1.29)	0.383		

*No condom use with last non-regular partner*				0.665		
No	29	36.0	1.00		---	
Yes	32	40.4	1.20 (0.52–2.78)	0.665		

*Ever STI symptoms*				0.491		
No	371	33.4	1.00			
Yes	31	29.1	0.82 (0.46–1.46)	0.491		

**Behavioural factors**	**HIV negative**					

*Number of sexual partners (past year)*				0.000		
None	652	13.6	1.00		1.00	
One	1992	35.6	3.50 (2.97–4.13)	0.000	1.71 (1.29–2.27)	0.000
Two or more	138	32.5	3.06 (2.16–4.33)	0.000	1.60 (1.04–2.45)	0.032

*No condom use at last sex*				0.000		
No	768	34.8	1.00		1.00	
Yes	841	45.8	1.59 (1.24–2.02)	0.000	1.58 (1.23–2.02)	0.000

*No condom use with last non-regular partner*				0.405		
No	141	38.7	1.00			
Yes	186	34.5	0.83 (0.55–1.28)	0.405	---	

*Ever STI symptoms*				0.609	---	
No	2366	26.3	1.00			
Yes	104	28.2	1.10 (0.77–1.57)	0.609		

^1^adjusted for sex and age

Bivariate analyses with HIV knowledge and risk variables (adjusted for sex and age) and knowledge of HIV status found among HIV positive persons that awareness of a place nearby where one could be tested for HIV, higher knowledge score on ARVs, knowledge of serodiscordance, impact of HIV on the household and high HIV risk perception were associated knowledge of HIV status, and among HIV negative persons awareness of a place nearby where one could be tested for HIV, high HIV knowledge score, knowledge of serodiscordance, impact of HIV on the household and high HIV risk perception were associated with knowledge of HIV status.

Multivariate analyses with HIV knowledge and risk variables (adjusted for sex and age) and knowledge of HIV status found among HIV positive persons that awareness of a place nearby where one could be tested for HIV, impact of HIV on the household and high HIV risk perception were associated knowledge of HIV status, and among HIV negative persons awareness of a place nearby where one could be tested for HIV, higher knowledge score on HIV and knowledge of serodiscordance were associated with knowledge of HIV status (see Table 4).

**Table 4:** Bivariate and multivarivate analyses of HIV knowledge and risk factors associated with knowledge of HIV status among HIV positive and negative persons

**HIV knowledge and risk variables**	**HIV positive**					
	N	Know HIV test result (%)	Crude OR (95% CI)	P-value	Adjusted OR (95% CI)^1 ^(Pseudo R square = .13)	P- value
*Aware of place nearby place to get tested for HIV*				0.000		
No	9	5.1	1.00		1.00	
Yes	426	38.6	11.7 (4.56–29.87)	0.000	8.85 (3.40–23.0)	0.000

*HIV knowledge*				0.092		
Low	102	29.4	1.00			
Medium	146	29.9	1.03 (0.66–1.59)	0.903		
High	179	37.0	1.41 (0.93–2.14)	0.107	---	

*Knowledge of ARVs*				0.420		
No	151	30.6	1.00			
Yes	283	33.6	1.15 (0.82–1.62)	0.420	---	

*Know of serodiscordance*				0.000		

*No*	180	27.4	1.00		1.00	
*Yes*	255	38.2	5.94 (3.52–10.04)	0.000	1.39 (0.96–1.99)	0.077

Impact of HIV (household: HIV, care death)				0.000		
No	342	28.2	1.00		1.00	
Yes	94	70.0	5.94 (3.52–10.04)	0.000	4.60 (2.54–8.32)	0.000

HIV risk perception				0.000		
Low	57	19.1	1.00		1.00	
Medium	115	29.1	1.74 (1.04–2.90)	0.034	1.38 (0.78–2.46)	0.269
High	254	39.4	2.75 (1.03–4.37)	0.000	1.71 (1.02–2.89)	0.042

AIDS stigma attitudes				0.997		
Low	192	32.9	1.00			
Medium	198	33.3	1.02 (0.72–1.44)	0.928	---	
High	41	32.4	0.98 (0.53–1.79)	0.939		

**HIV knowledge and risk variables**	**HIV negative**					

*Aware of place nearby place to get tested for HIV*				0.000		
No	92	4.1	1.00		1.00	
Yes	2682	32.9	11.6 (8.23–16.29)	0.000	9.73 (6.90–13.73)	0.000

*HIV knowledge*				0.000		
Low	601	17.1	1.00		1.00	
Medium	962	29.0	1.98 (1.64–2.39)	0.000	1.46 (1.18–1.80)	0.001
High	1181	32.2	2.30 (1.94–2.74)	0.000	1.64 (1.36–1.97)	0.000

*Knowledge of ARVs*				0.224		
No	938	25.3	1.00			
Yes	1838	27.1	1.10 (0.95–1.27)	0.224	---	

*Know of serodiscordance*				0.000		
*No*	951	20.1	1.00		1.00	
*Yes*	1823	32.0	1.87 (1.62–2.17)	0.000	1.56 (1.31–1.85)	0.000

Impact of HIV (household: HIV, care death)				0.024		
No	2660	26.1	1.00		1.00	
Yes	122	33.4	1.43 (1.05–1.94)	0.024	1.22 (0.86–1.73)	0.267

HIV risk perception				0.013		
Low	983	24.0	1.00		1.00	
Medium	924	27.1	1.17 (0.99–1.40)	0.071	1.05 (0.88–1.26)	0.600
High	870	28.3	1.25 (1.05–1.49)	0.014	0.96 (0.79–1.18)	0.711

AIDS stigma attitudes				0.237		
Low	1152	27.5	1.00			
Medium	1333	26.0	0.93 (0.80–1.08)	0.335	---	
High	273	25.1	0.88 (0.69–1.13)	0.324		

^1^Adjusted for sex and age

## Discussion

The study found from a large nationally representative population-based HIV survey that 27.6% had knowledge of their HIV status, and 7.8% of the total sample had taken an HIV test in the past 12 months and knew their test result. Similar to this finding, more than half of Southern African countries have less than 10% of their population who know their HIV status (in the past 12 months) [3]. Evaluations of traditional VCT systems suggest low uptake of VCT even in places where access to VCT is unlimited [1]. As access to ART increases, there is an urgent need for alternative VCT delivery systems to increase access to and the utilization of VCT. These alternatives include mobile VCT, routine offer of counselling and testing and home-based VCT. These models can increase access to and the uptake of VCT [24].

This study found in multivariate analysis that being female, the age group 25 to 34 years old, other than African Black population group (White, Coloured, Asian), higher educational level, being employed, urban residence, awareness of a place nearby where one could be tested for HIV, impact of HIV on the household and two or more sexual partners in the past year were associated with knowledge of HIV status. Other studies also found that socio-economic factors such as age and being female [5-9], educational level [6,10,11], occupation [7], area of residence [8], proximity and access to VCT site [6,9,12,16,17], knowledge of VCT sites [7,8] and HIV risk behaviour [7,8,10,11,18,19] were associated with knowledge of HIV status. Rural residence, African Black population group, lower educational level and being unemployed were significantly associated with not knowing one's HIV status; clearly efforts should be made to make HIV testing available and promote HIV testing among these people.

In this study HIV knowledge, knowledge about ARVs, HIV risk perception, HIV status, history of STI and AIDS stigma and discrimination were in multivariate analyses not found to be associated with knowledge of HIV status, unlike in findings from other studies where fear of unsolicited disclosure, fear of stigma and discrimination [1,6,7,9,12], marital status [8], HIV knowledge, HIV risk perception [7,8,10,11,18,19] and health status [5,7,8] were associated with knowledge of HIV status or HIV testing. The knowledge about ARVs was in this study not significantly related to knowing one's HIV status. The ART roll out in the public health sector had begun in South Africa in 2003, and one could have expected that more people get tested for HIV knowing about ARVs and thus accessing ART. It may be possible that in 2005 at the time of the survey antiretroviral treatment was not yet widely known and accessible; 39.5% of the survey respondents indicated that they had not heard about antiretroviral treatment.

The study found that among HIV positive persons awareness of a place nearby where one could be tested for HIV and impact of HIV on the household were associated knowledge of HIV status, and among HIV negative persons HIV risk behaviour (multiple partners, no condom use), awareness of a place nearby where one could be tested for HIV, higher knowledge score on HIV and knowledge of serodiscordance were associated knowledge of HIV status. The finding that HIV risk behaviour was associated with HIV test utilization among HIV negative persons seem to concur with the finding that VCT utilization is higher among low HIV risk groups [7,13]. With the increase in both educational level and knowledge of HIV/AIDS, accurate information about the disease and its causes and modes of transmission will be conveyed which seem to have led to HIV testing among HIV negative persons. In addition, sexual risk behaviour was associated with knowledge of HIV status among HIV negative persons, yet HIV risk perception was not found a predictor for knowledge of HIV status. The impact of HIV on the household was highly associated with knowledge of HIV status among HIV positive but not with HIV negative persons. Knowing someone living with or caring for someone with HIV and AIDS or someone who had died from AIDS was associated with knowledge of HIV status. With high rates of HIV in families and communities in South Africa there is an increased likelihood of knowing someone infected by the disease but it can also be that the respondent in these families are more likely to have been diagnosed with HIV themselves.

### Limitations

Caution should be taken when interpreting the results of this study due to certain limitations. Since this was a cross-sectional study, causality between the compared variables cannot be concluded. A further limitation was that a number of factors known to be contributing to knowledge of HIV status were not assessed, which included attitudes towards and desire for HIV testing [1,25]. Some measures in this study were limited in length, e.g. HIV risk perception and ARV knowledge was only measured with one item. The HIV testing and risk history measures did not include assessments of time since the behaviours occurred, not allowing us to examine whether people who were recently tested or who recently engaged in risk activities differed from those practising these behaviours less recently.

## Conclusion

In this setting, a disproportionate number of HIV-positive young, lower educated Black African rural men are failing to learn their status, which has implications for equitable access to onward referral for care and treatment services. Evidence that some high-risk behaviour may prompt HCT use is encouraging, although further interventions are required to improve knowledge about HIV risk and the benefits of HCT. Targeted interventions are also needed to promote HCT uptake among single, young and older persons and rural residents. For, example the use of mobile HCT (the provision of HIV counselling and testing services by mobile teams from a van equipped with HIV-testing facilities) can improve access for hard-to-reach and rural populations. The study findings further indicate that many persons in South Africa have never known their HIV status. Health-care providers should routinely screen all patients for HIV. New strategies such as mobile and home-based HCT are warranted to increase HIV testing, particularly among persons who are disproportionately affected by HIV infection.

## Competing interests

The authors declare that they have no competing interests.

## Authors' contributions

KP, GM, TM and MM conceptualized, analysed and interpreted the secondary data, drafted and revised the manuscript. All authors read and approved the final draft of the manuscript.

## Pre-publication history

The pre-publication history for this paper can be accessed here:


